# Navigating coeliac disease diagnosis in primary care

**DOI:** 10.3399/bjgp24X736137

**Published:** 2024-01-26

**Authors:** Alice M Harper, Jonathan Banks, Martha Elwenspoek, Deborah Lane, Kimberley Mousley, Mohamed G Shiha, Jessica Watson

**Affiliations:** Centre for Academic Primary Care, Population Health Sciences, Bristol Medical School, University of Bristol, Bristol.; NIHR Applied Research Collaboration (ARC) West; Population Health Sciences, Bristol Medical School, University of Bristol, Bristol.; NIHR ARC West; Population Health Sciences, Bristol Medical School, University of Bristol, Bristol.; Patient representative, Bristol.; Patient representative, Bristol.; Academic Unit of Gastroenterology, Sheffield Teaching Hospitals; Division of Clinical Medicine, School of Medicine and Population Health, University of Sheffield, Sheffield.; Centre for Academic Primary Care, Population Health Sciences, Bristol Medical School, University of Bristol, Bristol.

## Background

Coeliac disease (CD) is a common autoimmune condition that affects approximately 1% of the population worldwide.^1^ However, it is estimated that two thirds of patients with CD in the UK remain undiagnosed.^2^ CD can present at any age with a wide range of symptoms and signs, making recognition challenging.^3^ Traditionally, the diagnosis of CD requires serological testing followed by endoscopic biopsy while consuming a gluten-containing diet, as outlined in guidelines produced by the British Society of Gastroenterology (BSG) in 2014 and the National Institute for Health and Care Excellence (NICE) in 2015.^4^^,^^5^ There is variability in adherence to these guidelines in practice and clinical pathways are evolving.^6^

## Work-up in primary care

The work-up of CD in the UK usually begins in primary care. The first challenge is identifying patients with symptoms and risk factors of CD. Many CD symptoms are commonly seen in primary care and GPs need to be alert to the CD ‘clinical chameleon’.^3^ The second challenge is that current investigations require patients to be regularly consuming gluten – for instance, some gluten in more than one meal per day for at least six weeks before testing.^5^ Self-initiated gluten restriction without medical advice is increasingly common and dietary history is key. Discussing the re-introduction of gluten into the diet for testing may be difficult for GPs. Re-introduction of gluten may not be an option for some patients as this may cause severe and debilitating symptoms, and they should be referred to a gastroenterology specialist.^5^ Those with positive serology who consumed gluten during serological testing then enter an indeterminate diagnostic space where there is uncertainty about necessity and timeline for endoscopic biopsy.^7^ For some patients this presents an opportunity for self-diagnosis and self-management, to see if commencing a gluten-free diet (GFD) has a beneficial impact. Although guidance advises continuing a gluten-containing diet until diagnosis is confirmed by a specialist,^4^^,^^5^ given current delays in endoscopy, GPs and patients have to navigate this mid-diagnostic phase together.

## Guidelines and local pathways

Due to progress in serological testing and restricted access to endoscopy services exacerbated by the COVID-19 pandemic, the BSG published interim guidance for adults in 2020 that those aged <55 years with symptoms consistent with CD and no alarm symptoms can be diagnosed without endoscopic biopsy if immunoglobulin A (IgA) tissue transglutimase (tTG) is >10 times the upper limit of normal and a further IgA endomysial antibodies (EMA) test is positive.^8^^,^^9^ This interim guidance is stated as specific to the COVID-19 environment pending publication of the new BSG coeliac guideline. Given COVID-19 no longer constitutes a public health emergency and endoscopy services have resumed, there is uncertainty about what pathway GPs should be following and variation in how integrated care boards have interpreted guidance. Recent review of pre-pandemic cases has shown that a third of patients with positive IgA-tTG titres were not referred for an endoscopic biopsy despite guidance at the time advocating it.^10^ It is important for GPs to be aware that a non-biopsy approach is only for those with a high IgA-tTG titre, rather than any positive serology.

## Impact on patients

Challenges faced in primary care and variation in local diagnostic pathways will undoubtedly impact patients. We need to ensure we are identifying and working up patients with suspected CD to reduce underdiagnosis. On average, coeliac patients suffer symptoms for 13 years before getting diagnosed and starting a GFD.^11^ In our recent study, initial investigations for CD were often delayed because the non-specific symptoms had either been normalised by patients or attributed to alternative diagnoses by GPs.^7^ Quality of life is substantially lower in undiagnosed patients with CD compared to the general population, but improves on a GFD.^11^ Continued gluten exposure leads to accumulating damage of the intestinal lining and insufficient nutritional absorption, increasing the risk of anaemia, osteoporosis, and malignancy.^12^^–^^15^ We also need to recognise the potential for misdiagnosis in primary care. A reliance on self-reported gluten sensitivity to diagnose CD is often misleading due to substantial nocebo effect in people with non-coeliac gluten sensitivity.^16^ If this cohort follow a GFD, they may experience a short-lived improvement in symptoms but it creates a challenge when trying to formally diagnose potential cases of CD. A GFD is not recommended for people without CD because it is restrictive, costly, and could lead to nutritional deficiencies.^17^ Many processed gluten-free products have higher levels of fats, sugars, and salts, and a GFD may increase the risk for metabolic syndrome. On the other hand, patients who do have CD but do not receive a formal diagnosis may not: receive specialist dietary advice and support; appreciate implications for first-degree relatives; appreciate long-term complications and comorbidities; have an annual review including assessment of bone health; and receive appropriate immunisations. Without this information and support, such patients may have decreased adherence to their GFD and poorer outcomes.

## Impact on primary care

GPs need to be aware of the potential harms of suggesting a GFD to patients before they are diagnosed and should inform patients about the risks of trialling a GFD before diagnostic tests are completed – for example, symptoms in response to gluten might become more severe once on a GFD. There is a need for clear guidance for GPs on what to advise patients about their diet during CD testing, including harms of starting a GFD versus harms of continuing to eat gluten. Such guidance will facilitate personalised conversations and joint decision making. Future guidance should also clearly define which patients are eligible for a no-biopsy diagnosis. This needs to be adopted nationwide, and local protocols need to reflect this. More evidence may be needed to inform guidance relevant to primary care as current evidence for a no-biopsy approach is from secondary and tertiary care settings with a significantly higher prevalence of CD than you would expect in primary care.^6^^,^^9^^,^^18^ If future evidence supports that diagnosis of certain cases is transferred into primary care, healthcare professionals would have to be competent discussing accuracy of serological tests in order to empower patients to feel confident in their diagnosis and commitment to a life-long GFD. Appropriate follow up must also be available, including referral to a dietitian for all patients and referral to a gastroenterology specialist for those with lingering symptoms. The evolution of CD diagnostic guidance and clinical pathways will undoubtedly impact primary care – therefore, a close collaboration between primary and secondary care is needed to establish clear referral pathways and to provide patients with timely and appropriate care.
